# Distinct Regulatory Functions of Calpain 1 and 2 during Neural Stem Cell Self-Renewal and Differentiation

**DOI:** 10.1371/journal.pone.0033468

**Published:** 2012-03-14

**Authors:** Daniela M. Santos, Joana M. Xavier, Ana L. Morgado, Susana Solá, Cecília M. P. Rodrigues

**Affiliations:** 1 Research Institute for Medicines and Pharmaceutical Sciences (iMed.UL), Faculty of Pharmacy, University of Lisbon, Lisbon, Portugal; 2 Department of Biochemistry and Human Biology, Faculty of Pharmacy, University of Lisbon, Lisbon, Portugal; City of Hope National Medical Center and Beckman Research Institute, United States of America

## Abstract

Calpains are calcium regulated cysteine proteases that have been described in a wide range of cellular processes, including apoptosis, migration and cell cycle regulation. In addition, calpains have been implicated in differentiation, but their impact on neural differentiation requires further investigation. Here, we addressed the role of calpain 1 and calpain 2 in neural stem cell (NSC) self-renewal and differentiation. We found that calpain inhibition using either the chemical inhibitor calpeptin or the endogenous calpain inhibitor calpastatin favored differentiation of NSCs. This effect was associated with significant changes in cell cycle-related proteins and may be regulated by calcium. Interestingly, calpain 1 and calpain 2 were found to play distinct roles in NSC fate decision. Calpain 1 expression levels were higher in self-renewing NSC and decreased with differentiation, while calpain 2 increased throughout differentiation. In addition, calpain 1 silencing resulted in increased levels of both neuronal and glial markers, β-III Tubulin and glial fibrillary acidic protein (GFAP). Calpain 2 silencing elicited decreased levels of GFAP. These results support a role for calpain 1 in repressing differentiation, thus maintaining a proliferative NSC pool, and suggest that calpain 2 is involved in glial differentiation.

## Introduction

Differentiation is the process by which stem cells give rise to committed and specialized cells [1]. Stem cells have been successfully used in regenerative medicine [2],[3]. Nevertheless, the potential of stem cells is yet far from being fully explored and requires a better understanding of stem cell biology. Neural stem cells (NSC) have the ability to proliferate and self-renew, as well as to differentiate, following induction, into several neural cell types, including neurons, oligodendrocytes and glial cells [4]–[6]. Although thoroughly studied, the molecular pathways regulating differentiation of stem cells are still not fully defined, and may implicate cell cycle, apoptosis and migration, among other processes.

Calpains are a large conserved family of cysteine proteases regulated by calcium which cleave many different substrates, modulating protein activity [7]. Calpains have been implicated in the regulation of a wide range of cellular processes, including cell cycle, migration, apoptosis, autophagy and synaptic plasticity [8]–[14]. Calpain activity can be modulated by calcium and phospholipid binding, phosphorylation, autolysis and subcellular localization [7], [15], [16]. Additionally, calpains are regulated by the specific endogenous inhibitor calpastatin [17]. Unlike other proteases, calpains do not have a consensus substrate-binding or cleavage site, making it difficult to predict their possible substrates. In fact, substrates cleaved by different calpains vary depending on the context, probably as a consequence of the complex regulatory network affecting these proteases. Calpain 1 and calpain 2 are the most studied and abundant calpain molecules in the brain [8].

Although calpains have several important physiological functions, most of the studies involving these proteases in the central nervous system are disease-related. In fact, calpains have been implicated in several brain pathologies, such as Parkinson's disease, Alzheimer's disease, Huntington's disease, stroke and brain trauma [8], [18], [19]. The importance of calpains in synaptic function and memory formation has also been studied [8], [9].

Several studies have implicated calpains in differentiation mechanisms, including differentiation of mesenchymal stem cells, such as myoblasts, osteoblasts, chondrocytes and adipocytes [20]. A role for cysteine proteases in differentiation of embryonic stem cells into neural cells has also been reported, although the involvement of calpains in embryonic stem cell differentiation was argued against [21]. Nevertheless, neural progenitor cells exhibit calcium transients during cell cycle progression that are required for proliferation in cellular models [22]. In addition, mitogen-activated protein kinase (MAPK) and phosphatidylinositol-3 kinase (PI3K)/Akt are major signaling pathways implicated in a wide range of cellular processes [23], [24], including neural stem cell proliferation and differentiation [25]–[29]. These pathways have previously been implicated in regulation of calpain activity [15], [16], [30], [31]. Interestingly, calpain and calpastatin activities are modulated during neural differentiation of rat pheochromocytoma (PC12) cells [32]–[34]. Altered expressions levels for both calpain and calpastatin proteins were also described during human neuroblastoma cell differentiation to Schwann and neuronal cells [35]. Nevertheless, the potential function of calpains during neural differentiation is still poorly understood and requires further investigation.

In the present study, we elucidated the role of calpain 1 and 2 during NSC self-renewal and differentiation. Our results suggest that calpain 1 maintains stemness and represses neural differentiation. In addition, calpain 2 acts as potential modulator of gliogenesis. These results underscore the distinct regulatory functions of calpain 1 and 2 in NSC fate decision.

## Results

### Calpain inhibition decreases proliferation of neural stem cells

Although calpains have already been implicated in several differentiation systems [20], [32]–[34], their involvement during neural differentiation has not yet been fully explored. We have previously shown that cysteine proteases, such as caspases, regulate mouse NSC differentiation by interfering with the FOXO3A/Id1 signaling pathway [36].

**Figure 1:**
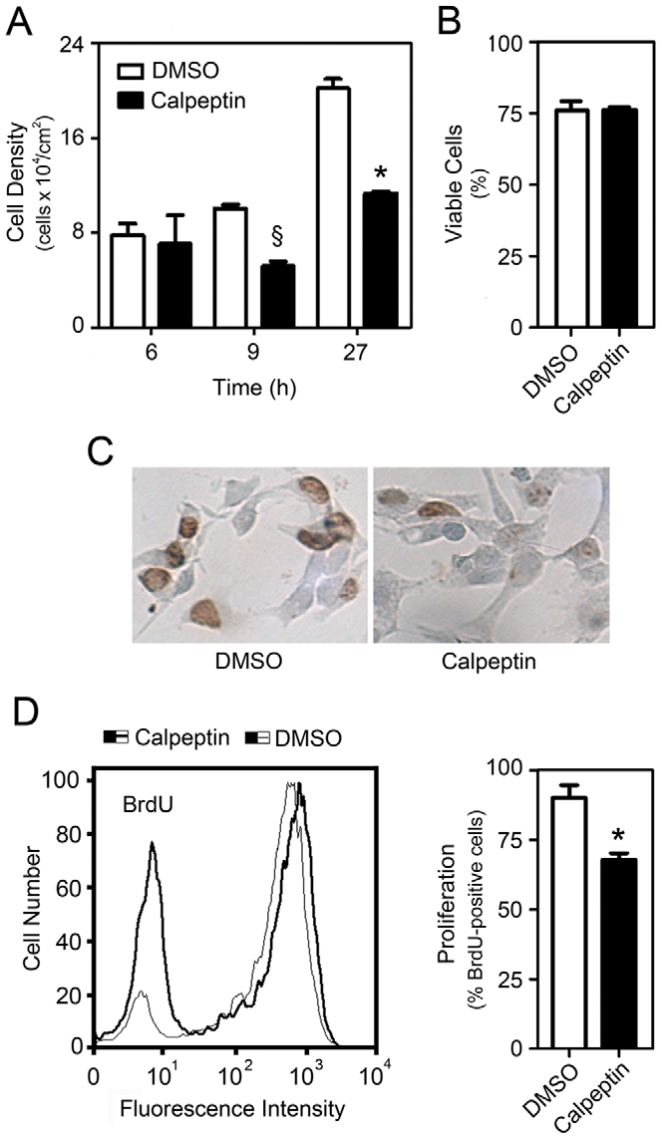
Calpeptin treatment decreases cell density and proliferation. NS-TGFP cells were cultured in permissive conditions and treated with either 50 µM calpeptin or DMSO (control), and BrdU was added 3 h later as described in *Materials and Methods*. (*A*) Cell density was accessed at different times after treatment and calculated as the number of live cells per cm^2^ dish surface area. (*B*) Cell viability was determined by flow cytometry after 24 h and expressed as the percentage of PI and Annexin-V double-negative cells. (*C*) Immunocytochemistry detection of BrdU-positive nuclei (brown). (*D*) Representative histogram of BrdU positive cells assessed by flow cytometry (left), and respective quantification data (right). Data represent mean ± SEM of three independent experiments. §*p*<0.05 and *p<0.01 from respective control.

To address the role of calpains in NSC self-renewal and differentiation, we first incubated NS-TGFP cells with the calpain chemical inhibitor calpeptin in permissive condition medium. This specific condition maintains cells in a proliferative status, although low levels of differentiation can be observed after a few days in culture. After 24 h, cells were treated with 50 µM calpeptin or dimethyl sulfoxide (DMSO; control) and incubated for additional 6, 9 or 27 h. Our results show that calpeptin treatment resulted in a marked decrease in cell number, eliciting almost 50% reduction in cell density at 27 h (*p*<0.01) (Figure 1A). However, this did not correlate with increased cell death, as measured by propidium iodide (PI) and Annexin-V staining, indicating low levels of toxicity (Figure 1B). Bromodeoxyuridine (BrdU) incorporation, in turn, revealed a 25% decrease in proliferation after calpeptin treatment, as assessed by immunocytochemistry (Figure 1C) and flow cytometry (Figure 1D) analysis (*p*<0.01).

**Figure 2:**
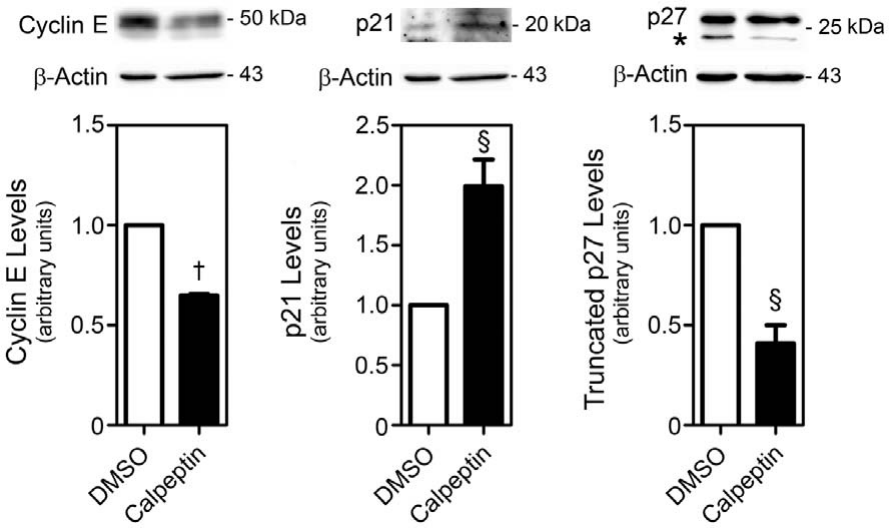
Calpeptin treatment modulates cell cycle proteins. NS-TGFP cells were cultured in permissive conditions, treated with either 50 µM calpeptin or DMSO (control) for 24 h and processed for immunoblotting as described in *Materials and Methods*. Representative immunoblots (top) and corresponding densitometry analysis (bottom) of cyclin E, p21 and p27 levels after calpeptin treatment. Two distinct p27 bands were detected, corresponding to full length (∼27 kDa) and truncated (marked with *) forms. Data represent mean ± SEM of three independent experiments. §*p*<0.05 and †*p*<0.001 from respective control.

To investigate whether inhibition of proliferation by calpains was due to altered cell cycle dynamics, we investigated the expression levels of several cell cycle markers. Interestingly, truncated p27 was decreased after calpeptin treatment of NS-TGFP cells (Figure 2). Truncation of cyclin-dependent kinase inhibitor p27 results in a substantial reduction in its inhibitory activity [37], [38]. In addition, calpain inhibition increased p21 and decreased cyclin E levels, indicative of impaired G1 progression in these conditions [39]. Our results suggest that calpain activity is involved in cell cycle progression and proliferation of NSC.

### Calpain inhibition increases differentiation of NSC

**Figure 3:**
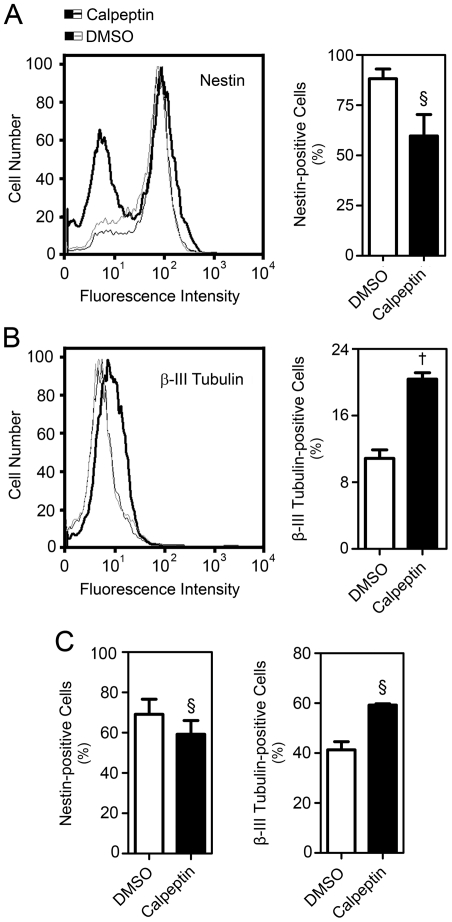
Calpeptin treatment decreases Nestin- and increases β-III Tubulin-positive cells. NS-TGFP cells and MNSC were cultured in permissive or differentiation conditions, respectively, treated with either 50 µM calpeptin or DMSO (control), and collected after 24 or 48 h as described in *Materials and Methods*. Cells were subsequently labeled for Nestin and β-III Tubulin detection by flow cytometry as described in *Materials and Methods*. (*A*) Representative histogram of Nestin-positive cells (left) and respective quantification data (right) in NS-TGFP cells after 24 h. (*B*) Representative histogram of β-III Tubulin-positive cells (left) and respective quantification data (right) in NS-TGFP cells after 24 h. (*C*) Quantification of Nestin- and β-III Tubulin-positive cells in MNSC after 24 or 48 h, respectively. Data represent mean ± SEM of at least three independent experiments. §*p*<0.05 and †*p*<0.001 from respective control.

Cell cycle dynamics can influence differentiation, including neurogenesis [39], [40]. We next investigated the differentiation status of calpeptin-treated NS-TGFP cells. Interestingly, calpeptin treatment decreased the proportion of Nestin-positive cells by ∼35% (*p*<0.05) (Figure 3A), while increasing the percentage of β-III Tubulin-positive by almost 90% (*p*<0.001) (Figure 3B), indicating loss of stemness and induction of neural differentiation, respectively. Similar results were obtained in a mouse neurosphere model (MNSC). Calpeptin-treated MNSC showed a 15% decrease in the proportion of Nestin-positive cells (*p*<0.05) and a 45% increase in the percentage of β-III Tubulin-positive cells (*p*<0.05) (Figure 3C).

**Figure 4:**
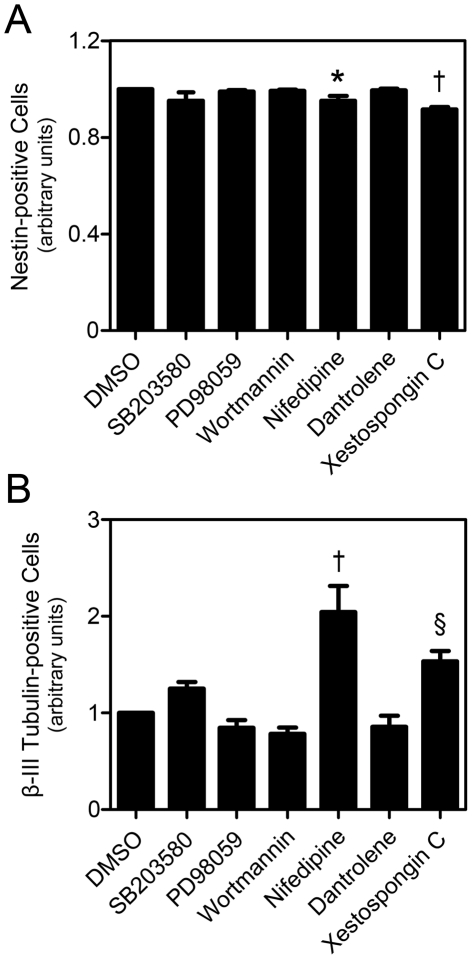
Inhibition of calcium flux decreases Nestin- and increases β-III Tubulin-positive cells. NS-TGFP cells were cultured in permissive conditions and treated with either 10 µM SB203580, 20 µM PD98059, 250 nM wortmannin, 20 µM nifedipine, 10 µM dantrolene, 1 µM xestospongin C or DMSO (control), and collected after 24 h. Cells were subsequently labeled for Nestin and β-III Tubulin detection by flow cytometry as described in *Materials and Methods*. (*A*) Quantification of Nestin-positive cells in NS-TGFP cells after different treatments. (*B*) Quantification of β-III Tubulin-positice cells. Data represent mean ± SEM of at least three independent experiments. §*p*<0.05, *p<0.01 and †*p*<0.001 from respective control.

We next sought to determine if any of the MAPK and PI3K/Akt pathways regulated calpain activity in NSC and if their inhibition mimicked the effects observed after calpain inhibition by calpeptin. NS-TGFP cells were treated with p38, extracellular signal-regulated kinase mitogen-activated protein kinase (MEK/ERK) and PI3K/Akt chemical inhibitors (SB203580, PD98059 and wortmannin, respectively), and the differentiation status was evaluated after 24 h (Figure 4A and B). Surprisingly, no significant differences were seen in the percentage of Nestin- or β-III Tubulin-positive cells, as compared to the control, indicating that, in these conditions, neither the MAPK p38 or MEK/ERK pathways, nor the PI3K/Akt pathway are involved in the calpain-mediated regulation of self-renewal and differentiation of NSC.

We have also treated NS-TGFP cells with several inhibitors for calcium receptor/channels, previously shown to maintain calcium oscillations in NSC [22], namely nifedipine, an L-type channel blocker; dantrolene, a ryanodine receptor channel antagonist; and xestospongin C, an inositol trisphosphate receptor (IP_3_R) inhibitor. Interestingly, while dantrolene elicited no significant alterations, both nifedipine and xestospongin C treatment resulted in differences similar to calpeptin treatment (Figure 4A and B). In fact, nifedipine and xestospongin C induced a small, but significant decrease in the percentage of Nestin-positive cells (*p*<0.01 and *p*<0.001, respectively), and a marked increase in the proportion of β-III Tubulin-positive cells (*p*<0.001 and *p*<0.05, respectively). Thus, our results suggest that calcium oscillations mediated by IP_3_R and L-type channels are responsible for calpain activation, maintenance of self-renewal and repression of differentiation of NSC.

### Calpain inhibition throughout neural differentiation increases neurogenesis

**Figure 5:**
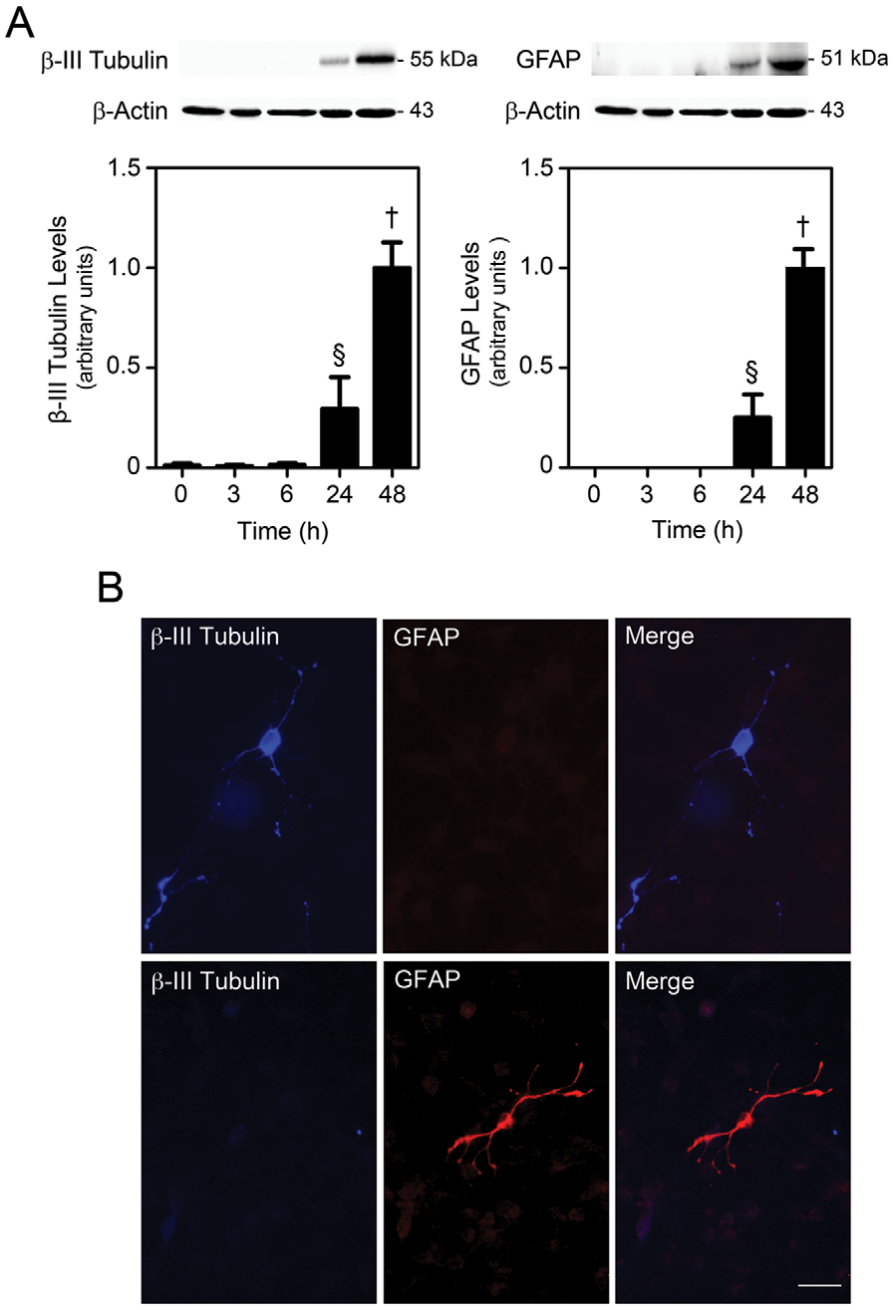
Neuronal and astrocytic phenotypes after induction of neural differentiation. β-III Tubulin and GFAP expression was evaluated in NS-TGFP cells at different times of differentiation as described in *Materials and Methods*. (*A*) Representative immunoblots (top) and corresponding densitometry analysis (bottom) showing an increase in β-III Tubulin and GFAP protein levels throughout neural differentiation. Results are expressed as mean ± SEM arbitrary units for at least three independent experiments. §*p*<0.05 and †*p*<0.001 from undifferentiated cells. (*B*) Immunofluorescence detection of neuronal and astrocytic morphology in cells co-stained with anti-β-III Tubulin and anti-GFAP antibodies after 4 days of neural differentiation. Scale bar, 20 µm.

To further explore the mechanism by which calpains regulate neural differentiation, we transfected NS-TGFP cells with calpastatin, an endogenous specific calpain inhibitor [17], and then induced neural differentiation. Cells were grown under differentiation conditions for 48 h and the expression of neuronal and glial differentiation markers β-III Tubulin and glial fibrillary acidic protein (GFAP) was accessed at several time-points. As expected, both neural markers increased throughout differentiation, starting at 24 h and peaking at 48 h (Figure 5A). Cells developed typical neuronal and astrocytic morphologies, accompanied by the expression of β-III Tubulin and GFAP, respectively, at 2 days (data not shown) and 4 days in culture (Figure 5B).

**Figure 6:**
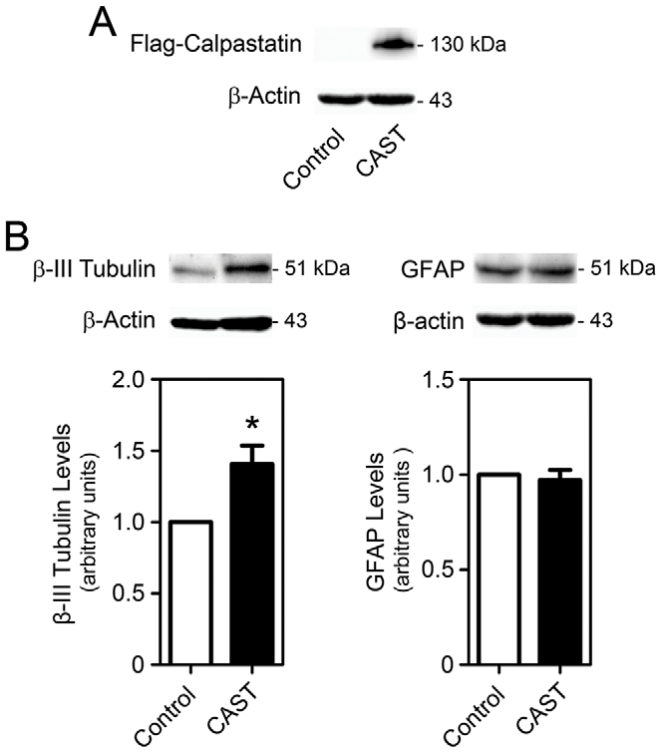
Calpastatin overexpression increases neuronal but not glial differentiation. β-III Tubulin and GFAP expression levels were evaluated by immunoblotting in NS-TGFP cells. Cells were transfected with either pcDNA-Flag-mCAST or pcDNA empty vector (control), differentiated and collected after 24 h, as described in *Materials and Methods*. (*A*) Representative immunoblots of Flag expression and β-actin in control and calpastatin overexpressing cells. (*B*) Representative immunoblots (top) and corresponding densitometry analysis (bottom) showing increased β-III Tubulin expression (left) and unchanged GFAP (right) protein levels in pcDNA-Flag-mCAST transfected cells. β-actin was used as loading control. Results are expressed as mean ± SEM arbitrary units for six independent experiments. *p<0.01 from control.

Following transfection, the Flag-calpastatin construct was easily detected by Western blot 24 h after induction of differentiation (Figure 6A). More importantly, calpastatin overexpression increased β-III Tubulin expression by 40% (*p*<0.05) (Figure 6B), corroborating our previous observation in calpeptin-treated NSC. However, no differences in GFAP levels were detected. Thus, our results reinforce the role of calpains in NSC fate and indicate that calpains may differently modulate neurogenesis and gliogenesis.

### Calpain 1 and 2 are differentially expressed throughout neural differentiation and do not correlate with cell death

**Figure 7:**
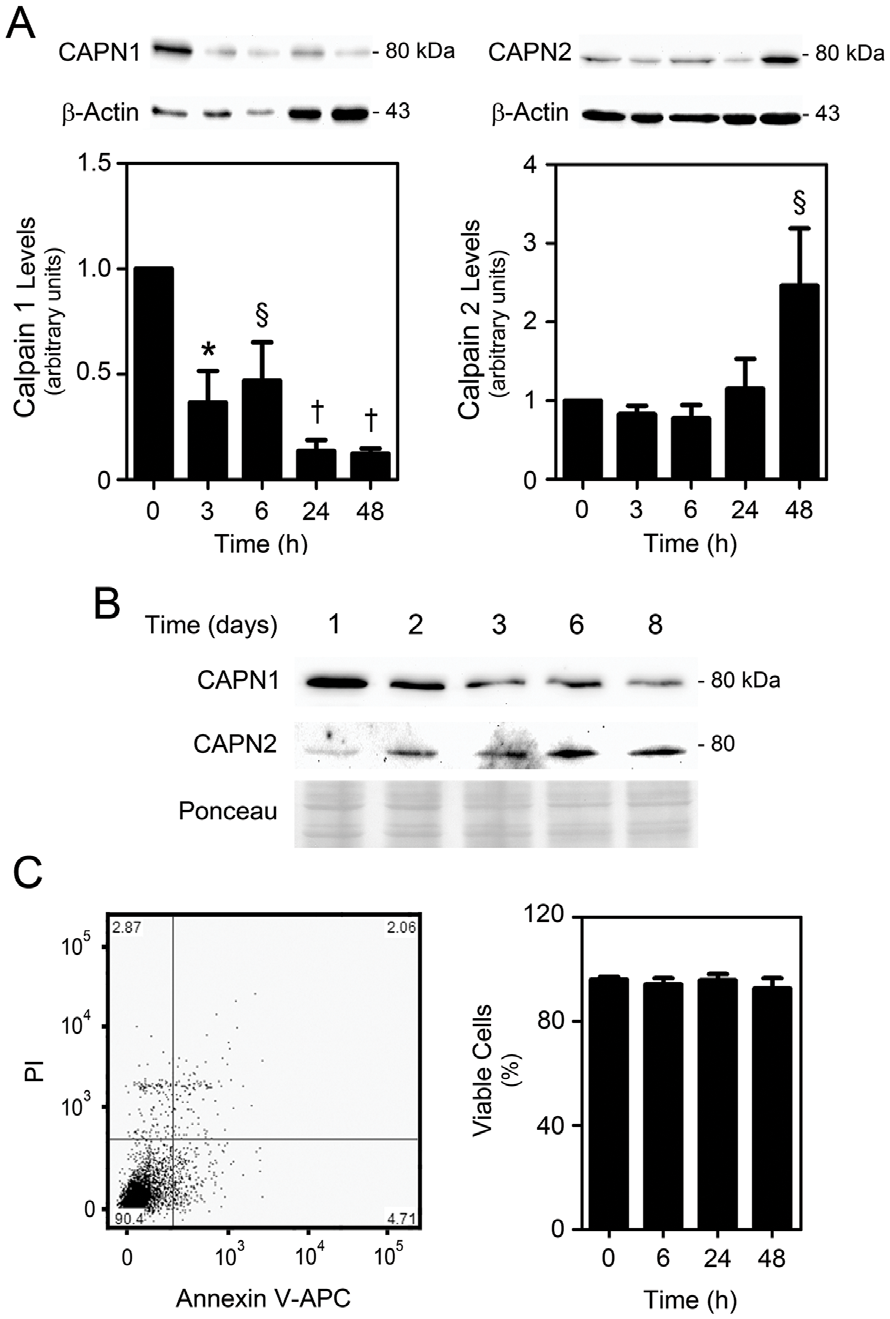
Calpain expression levels are altered throughout differentiation of NSC. NS-TGFP and MNSC were grown under differentiation conditions and collected for calpain 1 and 2 immunoblotting or stained with Annexin-V-APC/PI to evaluate cell death as described in *Materials and Methods*. (*A*) Representative immunoblots (top) and corresponding densitometry analysis (bottom) of calpain 1 and calpain 2 protein levels throughout NS-TGFP differentiation. β-actin was used as loading control. Results are expressed as the mean ± SEM arbitrary units for four independent experiments. §*p*<0.05, *p<0.01 and †*p*<0.001 from undifferentiated cells. (*B*) Representative immunoblots of calpain 1 and 2 expression throughout MNSC differentiation. Ponceau staining was used as loading control. (*C*) Representative Annexin-V-APC/PI data plot (left) and quantification data of viable (PI-negative, Annexin-V-negative) cells (right), showing absence of cell death throughout NS-TGFP differentiation. Data represent mean ± SEM of three independent experiments.

Calpain 1 and 2 are the most abundant calpain molecules in the brain [8], and calpastatin inhibits the activity of both proteins [17]. We next addressed their specific contributions to neural differentiation. Curiously, expression levels of calpain 1 were markedly higher in self-renewing NSC, and decreased significantly during differentiation of NS-TGFP cells (Figure 7A). In contrast, calpain 2 increased throughout differentiation in a similar manner as β-III Tubulin and GFAP. In MNSC, β-III Tubulin- and GFAP-positive cells have previously been detected at 3 and 8 days of differentiation, respectively [36]. Accordingly, the expression patterns of both calpain 1 and 2 were similar to those found in NS-TGFP undergoing differentiation, with levels of calpain 1 decreasing as calpain 2 increased throughout neural differentiation (Figure 7B). Thus, our results suggest that calpain 1 and 2 have distinct functions throughout NSC differentiation. Finally, as calpains are apoptosis-associated proteases, we searched for a correlation between calpain expression and cell death throughout differentiation. We have previously shown that differentiation of MNSC was not associated with an increase in cell death [41]. Similarly, our results reveal no significant differences in viability and cell death of differentiating NS-TGFP cells, as measured by PI and Annexin-V staining (Figure 7C). These results indicate that calpains may be important for the regulation of neural differentiation in a cell death-independent manner.

### Calpain 1 represses neural differentiation, while calpain 2 increases glial differentiation

**Figure 8:**
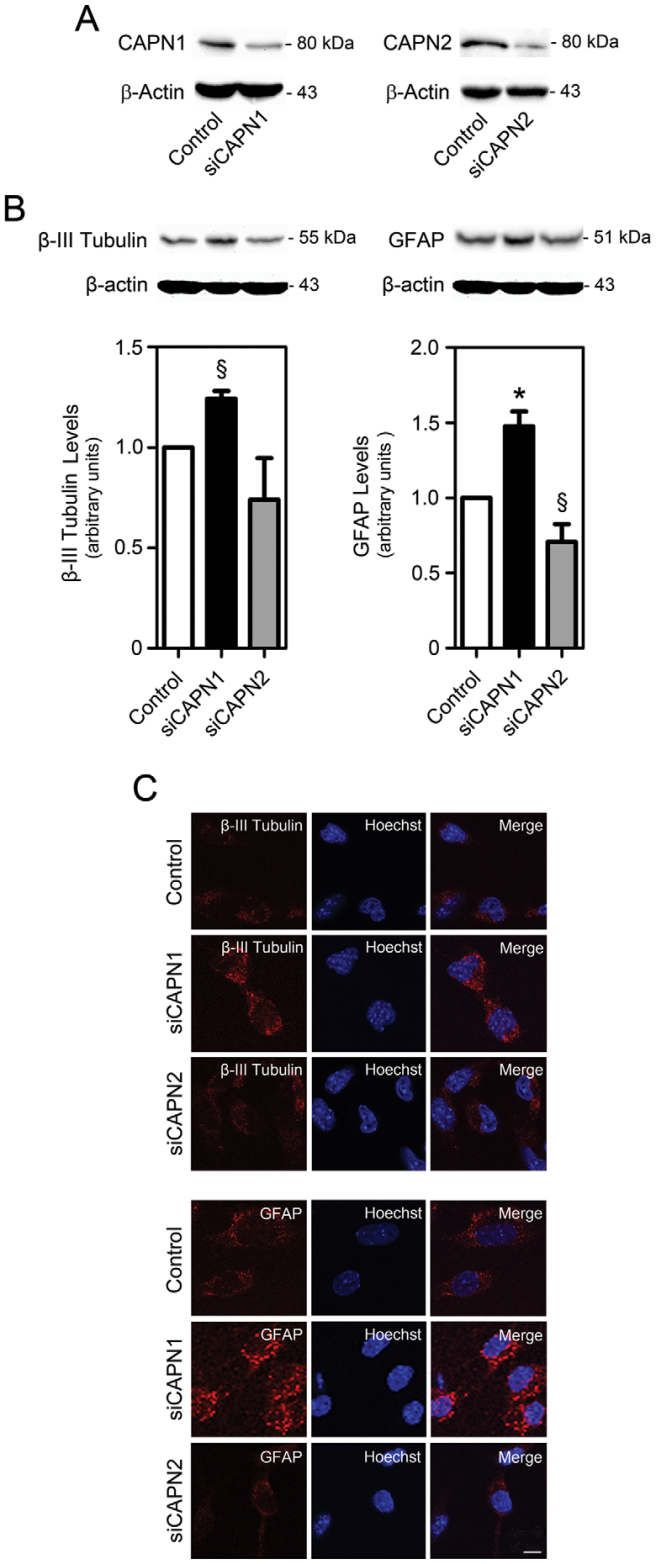
Calpain 1 and 2 silencing regulates the expression of neural differentiation markers. NS-TGFP cells were transfected with siRNAs for either calpain 1 (siCAPN1), calpain 2 (siCAPN2) or control siRNA, and then differentiated. Cells were fixed for immunocytochemistry or collected for Western blot analysis as described in *Materials and Methods*. (*A*) Representative immunoblots showing a reduction of calpain 1 (left) and calpain 2 (right) protein levels after siRNA-induced silencing. (*B*) Representative immunoblots (top) and corresponding densitometry analysis (bottom) showing altered β-III Tubulin (left) and GFAP (right) protein levels following calpain 1 or calpain 2 silencing. Results are expressed as mean ± SEM arbitrary units for at least three independent experiments. β-actin was used as loading control. §*p*<0.05 and *p<0.01 from control. (*C*) Confocal immunofluorescence detection of cells labeled with anti-β-III Tubulin and anti-GFAP antibodies shows increased expression of β-III Tubulin and GFAP following calpain 1 knockdown and decreased expression of GFAP following calpain 2 knockdown in differentiating NS-TGFP cells. Hoechst 33258 staining was used to visualize cell nuclei. Scale bar, 10 µm.

To further investigate the precise roles of calpain 1 and 2 during neural differentiation, we transfected NS-TGPF cells with siRNA specific for calpain 1 (siCAPN1) or calpain 2 (siCAPN2), or unspecific control, and then induced differentiation. Reduced expression of calpain 1 and 2 was observed by Western blot in cells treated with siCAPN1 and siCAPN2, respectively (Figure 8A). Both β-III Tubulin and GFAP expression levels were ∼25% (*p*<0.05) and 50% (*p*<0.01) increased after calpain 1 silencing (Figure 8B). Calpain 2 knockdown, in turn, elicited a significant decrease in GFAP expression (*p*<0.05), but no differences in β-III Tubulin expression. These results were corroborated by immunocytochemistry analysis (Figure 8C). The opposing actions of calpain 1 and 2 on GFAP expression may explain the absence of difference found earlier in GFAP upon calpastatin overexpression. Thus, our results suggest that calpain 1 plays a role in cell cycle progression of NSC, delaying differentiation and maintaining a proliferative neural stem cell pool, while calpain 2 may be important for glial differentiation.

## Discussion

The present study identifies a distinct regulatory function of calpains during NSC proliferation and differentiation *in vitro*. Calpain 1 represses both neuronal and glial differentiation, while calpain 2 is associated with glial differentiation.

Transplantation of stem cells may provide a more permanent remedy than present drug treatments for cell replacement in various neurodegenerative diseases. However, stem cells survive and differentiate poorly after transplantation [42], [43]. Curiously, it has been shown that several conserved elements of apoptosis are also integral components of terminal differentiation [44], [45], suggesting that apoptosis-related proteins might be important players of cell fate decisions. We have recently demonstrated the involvement of specific apoptosis-associated molecules in mouse NSC differentiation. In fact, apoptosis-associated miRNAs were involved in neural differentiation [41], and caspase inhibition and p53 silencing synergistically delayed neural differentiation, with no evidence of apoptosis [36]. Here, we investigated the potential role of proteases such as calpains in the regulation of NSC self-renewal and differentiation.

Much like caspases, calpains are cysteine proteinases that once activated cleave a wide range of cellular substrates [7]. Calpains are regulated by calcium and several studies have already demonstrated calpain involvement in differentiation systems, supporting the idea that apoptosis-associated factors are involved in regulation of the differentiation process. In fact, it has been demonstrated that during muscle cell differentiation, calpains relocate to the cell membrane of myoblasts in response to calcium flux and participate in fusion associated protein degradation [46], [47]. Calpains have also been implicated in osteoblast and chondrocyte differentiation [20], as well as in the turnover of transcriptional nuclear proteins driving differentiation of 3T3-L1 preadipocytes [48], [49]. However, calpain activation has not always been associated with promotion of cellular differentiation. The conversion of ST-13 preadipocytes into adipocytes was shown to be enhanced by calpain inhibition [20]. The specific role of calpains during neural differentiation remains largely unknown and requires further investigation, as only a few studies were performed in appropriate cell models [32]–[35].

In the present study, we first demonstrated that treatment of NS-TGFP mouse NSC with the calpain chemical inhibitor calpeptin decreases both cell number and proliferation. Our data is in accordance with other studies highlighting the importance of calpain activity in cell cycle progression, particularly in the transition from G1 to S phase [50]–[53]. We show that inhibition of calpain activity in NSC by calpeptin leads to a significant decrease in cyclin E levels, responsible for the G1/S transition. The impairment of cell cycle progression induced by calpeptin was also detected by p21 accumulation and decreased p27 degradation, evidenced by the presence of a lower molecular weight truncated fragment [38]. In fact, it has already been shown that deletion of calpain regulatory small subunit *Capn4* results in impaired cell cycle progression in chondrocytes. This specific deletion led to an accumulation of certain cell cycle proteins known as calpain substrates, such as cyclin D, cyclin E, and p27, as well as reduced phosphorylation of retinoblastoma protein and p27 [50]. p27 transcription and degradation, in turn, was also shown to be mediated by calpains in other models, including osteoblasts, cortical neurons and preadipocytes [51]–[53]. Further, p21 accumulation induced by calpain inhibition has also been observed in other studies, where *in vitro* incubation with calpain 1 and 2 resulted in rapid degradation of p21 [54],[55]. It has been demonstrated that calcium oscillations occurring in G1 to S transition are required for cell cycle progression in both neural progenitor and undifferentiated cells, correlating with G1 shortening and increased proliferation [22], [56]. Furthermore, calcium oscillations increase the levels of several proliferation-associated proteins and decrease p27-mediated inhibition [22]. Curiously, it was recently proposed that the length of G1 directly influences the differentiation rate of neural precursors [39], [56], [57]. In this respect, it appears that G1 phase prolongation is both necessary and sufficient to induce switching from proliferation to differentiation in neural progenitors [39]. Therefore, it is not surprising that calpains accelerate G1 to S transition and that calpain inhibition is necessary to increase G1 length and promote neural stem cell differentiation. Consistent with this hypothesis, our results revealed that treatment of neural stem cells with calpeptin induces a significant decrease in the proportion of Nestin-positive neural progenitors, while increasing the proportion of neuronal cells positive for β-III Tubulin. These results were also obtained in MNSC cultures, revealing that NSC differentiation induced by calpain inhibition was not restricted to the NS-TGFP cell line. To clarify the specific signaling pathway that regulate calpain activity in NSC, NS-TGFP cells were treated with different chemical inhibitors of major signaling pathways. Our results showed that, similarly to calpeptin treatment, inhibition of calcium flux strongly influences NSC fate decision. We tested the IP_3_R, L-type channel and ryanodine receptor inhibitors xestospongin C, nifedipine, and dantrolene, respectively, and observed that the first two had a significant effect in NSC differentiation, decreasing Nestin and increasing β-III Tubulin positive cells in the population. In fact, it has already been shown that calcium oscillations in neural progenitor cells are restricted to the G1/S transition and require calcium influx only through IP_3_Rs, L-type channels and ryanodine receptors [22]. Since calpains are calcium-activated proteases, it is possible that calpains are regulated by calcium oscillations in G1, thereby promoting cell cycle progression through modulation of cell cycle proteins.

To further address the role of calpains during neural differentiation, the expression levels of specific calpain 1 and 2 were investigated and found to be strikingly different throughout NSC differentiation. In fact, while calpain 1 expression was higher during self-renewal and decreased throughout differentiation, expression of calpain 2 markedly increased during neural differentiation in both NS-TGFP and MNSC. More importantly, fluctuations in calpain expression are not associated with cell-death signaling pathways, as no difference was detected in cell viability throughout differentiation in both cell models. Thus, our observation raises the possibility that calpain 1 and 2 differentially regulate NSC biology. The fact that calpain 1 is mostly expressed in self-renewing NSC suggests its involvement in regulation of proliferation mechanisms. Indeed, distinct functions for both calpains have already been reported. Calpain 1, but not calpain 2, was shown to be present in the nucleus and cytoplasm of cultured cortical neurons, being capable of degrading p27 in cell lysates [52]. In addition, calpain 1 degraded the G1 cyclin dependent kinase inhibitor p19^INK4d^ *in vitro* [58], consistent with a role in the regulation of cellular self-renewal. Notably, our data validates this hypothesis by showing that calpain 1 silencing increases both neuronal and glial differentiation.

In this study, calpain 2 levels increased throughout neural differentiation, suggesting that this specific calpain plays a role later in differentiation. Accordingly, studies on PC12 neuronal differentiation have demonstrated that although calpain inhibition is important in early events of the differentiation process, calpains may be required in later stages as well [32], [34]. In fact, calpastatin levels declined later during differentiation in a caspase-1 dependent manner, allowing calpain-mediated cleavage of fodrin [34]. Our results showed that calpain 2 silencing elicited a significant decrease in GFAP expression during neural differentiation, while no significant differences were detected in β-III Tubulin expression. This finding, coupled with others showing that calpain 2 is mostly localized in glial cells, while calpain 1 is located primarily in neurons [52], [59], [60], suggests that calpain 2 activity is important for glial, but not neuronal differentiation. In fact, the inhibition of both calpains during neural differentiation by calpastatin resulted in a marked increase in β-III Tubulin expression and no differences in GFAP levels. If calpain 1 is indeed responsible for maintaining NSC self-renewal, it would be expected that its inhibition would induce a significant increase in both β-III Tubulin and GFAP, as detected after calpain 1 silencing. However, it is not surprising that GFAP levels remained unchanged after calpastatin treatment if we consider that inhibition of calpain 2 leads to decreased GFAP expression. Thus, calpain regulation of neural stem cell fate choices is apparently a complex process that may involve a tightly coordinated action of different calpains during self-renewal and differentiation stages.

Collectively, our results support a role for calpain 1 in the maintenance of a proliferative neural stem cell pool, and suggest that calpain 2 is involved in the onset of glial differentiation. Further insight into the factors regulating calpain 1 and 2 activities and their specific substrates during neural differentiation is necessary to understand the complexity of calpain function in NSC fate decision.

## Materials and Methods

### Ethics statement

The embryonic stem-derived mouse NSC line, a Tau- green fluorescent protein cell line (NS-TGFP) was obtained from Dr. Smith's Laboratory, University of Cambridge, Cambridge, UK [61], and provided by Dr. Henrique, University of Lisbon, Lisbon, Portugal. Neurospheres of MNSC were obtained from Dr. Reynold's Laboratory, University of Queensland, Brisbane, Australia, and provided by Dr. Low, University of Minnesota, Minneapolis, MN, USA. The Animal Ethical Committee at the Faculty of Pharmacy, University of Lisbon, Portugal waived the need for approval.

### Cell culture and treatments

NS-TGFP cells were derived from 14.5-days post coitum mouse fetal forebrain, and constitutively express the fusion protein Tau-GFP [61], [62]. This cell line was established using a method that produces pure cultures of adherent NSC, which continuously expand by symmetrical division and are capable of tripotential differentiation [63]–[65]. NS-TGFP cells were grown in monolayer as previously described [66] and routinely maintained in undifferentiation medium, Euromed-N medium (EuroClone S.p.A., Pavia, Italy), supplemented with 1% N-2 supplement (Invitrogen Corp., Grand Island, NY), 20 ng/mL epidermal growth factor (EGF; PeproTech EC, London, UK), 20 ng/mL basic fibroblast growth factor (bFGF; PeproTech EC) and 1% penicillin-streptomycin (Invitrogen Corp.), in uncoated tissue culture plastic flasks at 37°C in a humidified atmosphere of 5% CO_2_. Medium was changed every 3 days and cells collected with accutase (Sigma-Aldrich Co., St. Louis, MO) when confluent. Permissive conditions were obtained by platting NSC in tissue culture plates pre-coated with 0.1% gelatin (Sigma-Aldrich Co.) at 3×10^4^ cells/cm^2^ in N2B27 medium, 1∶1 mixture of DMEM/F12 (Invitrogen Corp.) and Neurobasal (Invitrogen Corp.), supplemented with 0.5% N-2 supplement, 1% B27 supplement (Invitrogen Corp.) and 2 mM L-Glutamine (Invitrogen Corp.). N2B27 medium was further supplemented with 10 ng/mL EGF, 10 ng/mL bFGF and 1% penicillin-streptomycin. After 24 h in permissive conditions, 50 µM calpeptin (Tocris Bioscience, Bristol, UK), 10 µM SB203580 (Tocris Bioscience), 25 µM PD98059, 250 nM wortmannin, 20 µM nifedipine, 10 µM dantrolene, 1 µM xestospongin C or DMSO (all from Sigma-Aldrich Co.) were added to the culture medium for 6, 9, 24 or 27 h. After collection with accutase, cells were counted and processed for flow cytometry analysis, BrdU staining or immunoblotting. Differentiation of NS-TGFP cells was performed by first platting cells in undifferentiation medium onto uncoated tissue culture plastic dishes at 3×10^4^ cells/cm^2^ for 24 h, and changing the culture medium to differentiation medium, Euromed-N medium supplemented with 10 ng/mL bFGF, 0.5% N-2 supplement, 1% B27 supplement and 1% penicillin-streptomycin. Cells were collected before medium change (time 0), or cultured for additional 3, 6, 24 or 48 h, and then collected for cell death analysis and protein extraction. For microscopic detection of neuronal and astrocytic morphology and for immunocytochemical co-labeling of β-III Tubulin and GFAP, cells were fixed at 2 days of differentiation or 4 days of differentiation in the presence of 1% fetal bovine serum (FBS) (Invitrogen Corp.) for the last 2 days in culture, and then processed as described below.

Primary MNSC containing a constitutively expressed marker for GFP were also used in selected experiments. MNSC were obtained from central nervous system tissue of embryonic mice [67]–[69], maintained as neurospheres and induced to differentiate as previously described [36], [41]. Cells were collected at 1, 2, 3, 6 or 8 days after induction of differentiation and processed for flow cytometry analysis or immunobloting assays.

### siRNA and plasmid transfections

For short interference RNA (siRNA) transfections, two pools of 4 siRNA nucleotides designed to knockdown mouse calpain 1 (L-062006-00-0005) and calpain 2 (L-043027-00-0005) expression were purchased from Dharmacon (Waltham, MA). A control siRNA containing a scrambled sequence that does not lead to the specific degradation of any known cellular mRNA was used as control. Briefly, cells were first cultured in uncoated dishes in undifferentiation medium without penicillin-streptomycin. Twenty four hours after plating, cells were transfected with 100 nM siRNA in the presence of 10% FBS using Lipofectamine 2000 (Invitrogen Corp.), according to the manufacturer's instructions. Six hours later, the medium was changed to differentiation medium and cells were cultured for additional 24 and 48 h. Efficiencies of calpain 1 and calpain 2 silencing were assessed by immunoblotting. Calpain inhibition was also achieved by overexpressing the endogenous inhibitor calpastatin. Briefly, cells were transfected with ∼2 µg pcDNA-Flag-mCAST construct, kindly provided by Dr. Duarte (Center for Neuroscience and Cell Biology, Coimbra, Portugal), or with pcDNA empty vector. The Flag-mCAST construct was prepared by cloning full-length mouse calpastatin cDNA with N-terminal Flag into pcDNA vector (Invitrogen Corp.). Cells were cultured in uncoated dishes in N2B27 medium supplemented with 10 ng/mL EGF and 10 ng/mL bFGF for 24 h, transfected for 6 h using Lipofectamine 2000 and then the medium was changed to differentiation medium. Calpastatin overexpression was evaluated by immunoblotting against the Flag tag.

### Flow cytometry analysis

NS-TGFP cells were washed twice with Ca^2+^- and Mg^2+^-free PBS (Invitrogen Corp.), treated with accutase and harvested with PBS. MNSC were trypsinized (0.025% trypsin/EDTA) (Invitrogen Corp.) and harvested in Ca^2+^-free and Mg^2+^-free PBS and 2% FBS. For cell death analysis, cells were stained with the vital dye PI (5 µg/mL; Sigma-Aldrich Co.) and Annexin-V-APC (eBioscience, Inc., San Diego, CA), according to manufacturer's instructions, to determine phosphatidylserine exposure. Proliferation levels were determined by BrdU incorporation analysis using the APC BrdU Flow Kit (BD Biosciences Pharmingen, San Diego, CA). BrdU was added to the culture medium 3 h after cell treatments, and cells were re-incubated for additional 6 h for flow cytometry analysis.

For detection of Nestin and β-III Tubulin expression levels, cells were fixed with paraformaldehyde (4% w/v) in PBS for 20 min at 4°C, washed twice with washing solution 0.1% saponin (Fluka, Biochemika, Switzerland) in PBS, and blocked for 20 min in blocking solution 0.25% saponin and 5% FBS in PBS. Subsequently, cells were washed and incubated with antibodies reactive to Nestin (MAB 353; Chemicon International, Temecula, CA) or β-III Tubulin (Tuj1; Covance, Princeton, New Jersey) at a dilution of 1∶300 and 1∶500, respectively, in antibody blocking solution (0.1% saponin and 5% FBS in PBS), for 30 min. Cells were then washed twice and incubated with anti-mouse antibody conjugated to Dylight 649 (Jackson ImmunoResearch Laboratories, Inc., West Grove, PA), at a dilution of 1∶5000 for 30 min. Cells were washed twice, resuspended in PBS with 2% FBS and analyzed using the FACSCalibur (Becton Dickinson, Mountain View, CA). Data were statistically evaluated using FlowJo software (Tree Star, Inc, Ashland, OR).

### Immunoblotting

Steady-state levels of cyclin E, p21, p27, β-III Tubulin, GFAP, calpain 1, calpain 2, Flag-calpastatin and β-actin were determined by immunoblotting. Cells were collected and lysed for isolation of total protein extracts with lysis buffer (50 mM KCl, 50 mM PIPES, 10 mM EGTA, 2 mM MgCl_2_, 0.5% Triton X-100, pH 7.4) supplemented with 100 µM PMSF, 1 mM DTT and Halt Protease and Phosphatase Inhibitor Cocktail (Thermo Fisher Scientific Inc., Rockford, IL), followed by centrifugation at 200*g* at 4°C for 20 min. Protein content was measured by the Bio-Rad protein assay kit (Bio-Rad Laboratories, Hercules, CA, USA) according to the manufacturer's specifications, using bovine serum albumin as standard. Fifty to one hundred µg of total protein extracts were separated on 8% sodium dodecyl sulphate-polyacrylamide electrophoresis gel, and then subjected to immunoblotting using primary mouse monoclonal antibodies reactive to p27 (Santa Cruz Biotechnology, Santa Cruz, CA), β-III tubulin (Tuj1; Covance), GFAP (MAB360; Chemicon International), Flag (M2; Sigma-Aldrich Co.) or β-actin (A5441; Sigma-Aldrich Co.), or primary rabbit polyclonal antibodies reactive to cyclin E (Santa Cruz Biotechnology), calpain 1 (sc-7531-R; Santa Cruz Biotechnology) or calpain 2 (2539; Cell Signaling Technology, Inc., Beverly, MA), or primary goat polyclonal antibodies reactive to p21 (Santa Cruz Biotechnology). Blots were subsequently incubated with secondary antibodies conjugated with horseradish peroxidase (Bio-Rad Laboratories). Finally, membranes were processed for protein detection using Immobilon (Millipore Corporation, Billerica, MA) or SuperSignal reagent (Pierce, Rockford, IL). Ponceau S staining was also used to assess equal gel loading.

### Immunocytochemistry

For detection of proliferating cells, S-phase nuclei were stained using the BrdU *In-Situ* Detection Kit (BD Biosciences Pharmingen). BrdU was added to the culture medium 3 h after cell treatments, and cells were re-incubated for additional 6 h and processed according to manufacturer's instructions. For fluorescence microscopy, NS-TGFP cells were fixed with paraformaldehyde (4%, w/v) in PBS and blocked for 1 h at room temperature in PBS, containing 0.1% Triton X-100, 1% FBS, and 10% normal donkey serum (Jackson ImmunoResearch Laboratories, Inc.). For single staining, cells were incubated with either anti-β-III Tubulin or anti-GFAP antibodies at a dilution of 1∶1000 in blocking solution, overnight at 4°C. Cells were then incubated with an Alexa 568-conjugated anti-mouse antibody (Life Technologies Ltd, Paisley, UK) at a dilution of 1∶200 in blocking solution, for 2 h at room temperature. Mouse NS cell nuclei were then stained with Hoechst 33258 (Sigma-Aldrich Co.) at 50 µg/ml in PBS, for 5 min at room temperature. For co-labeling of β-III Tubulin and GAFP, cells were incubated with mouse anti-β-III Tubulin and rabbit anti-GFAP (Sigma-Aldrich Co.) antibodies at a dilution of 1∶500 and 1∶250, respectively, in blocking solution, overnight at 4°C. Cells were then incubated with Alexa 405-conjugated anti-mouse and Alexa 594-conjugated anti-rabbit antibodies (Life Technologies Ltd) at a dilution of 1∶200 each, for 2 h at room temperature. Samples were mounted using Fluoromount-G™ (Beckman Coulter, Inc., Brea, CA). Fluorescence microscopy assessments were performed with a Zeizz AX10 microscope (Carl Zeiss, Jena, Germany) equipped with a Leica DFC490 camera (Leica Wetzlar, Germany) or with a Zeiss LSM 510 META confocal microscope (Carl Zeiss).

### Densitometry and statistical analysis

The relative intensities of protein bands were analyzed using the Quantity One Version 4.6.3 densitometric analysis program (Bio-Rad Laboratories). Results from different groups were compared using the Student's t test, two-way ANOVA or one-way ANOVA followed by Bonferroni's or Dunnett's multiple comparison tests. Values of p<0.05 were considered statistically significant. All statistical analysis was performed with GraphPad Prism 5 software (GraphPad Software, Inc., San Diego, CA).
